# Basal nucleation and the prevalence of ascending swarms in Long Valley caldera

**DOI:** 10.1126/sciadv.abi8368

**Published:** 2021-08-27

**Authors:** Bing Q. Li, Jonathan D. Smith, Zachary E. Ross

**Affiliations:** 1 Seismological Laboratory, California Institute of Technology, Pasadena, CA 91125, USA.; 2 Department of Civil and Environmental Engineering, Western University, London, ON, Canada.

## Abstract

Earthquake swarms are ubiquitous in volcanic systems, being manifestations of underlying nontectonic processes such as magma intrusions or volatile fluid transport. The Long Valley caldera, California, is one such setting where episodic earthquake swarms and persistent uplift suggest the presence of active magmatism. We quantify the long-term spatial and temporal characteristics of seismicity in the region using cluster analysis on a 25-year high-resolution earthquake catalog derived using leading-edge deep-learning algorithms. Our results show that earthquake swarms beneath the caldera exhibit enlarged families with statistically significant tendency for upward migration patterns. The ascending swarms tend to nucleate at the base of the seismogenic zone with a spatial footprint that is laterally constrained by the southern rim of the caldera. We suggest that these swarms are driven by the transport of volatile-rich fluids released from deep volcanic processes. The observations highlight the potential for extreme spatial segmentation of earthquake triggering processes in magmatic systems.

## Teaser

Long Valley caldera generates swarms at the base of the seismogenic zone with prevalent upward migration.

## INTRODUCTION

Earthquake swarms are a pervasive yet mysterious feature of magmatic systems, with a variety of nontectonic driving mechanisms including inflation of magma bodies (*1*, *2*), caldera collapse (*3*), dike intrusions (*4*), and degassing processes (*5*). In some instances, swarms have served as precursors to volcanic eruptions (*6*, *7*), while in other circumstances they are seemingly unrelated (*8*, *9*). Zobin (*10*) gives a detailed outline of case studies from many magmatic swarming regions. For example, there appears to be notable depth segmentation between deep (10 to 20 km) and shallow events, often separated by an aseismic zone corresponding to viscous material within or near magma chambers. This depth segmentation is also evident as a temporal segmentation, where the initial stages of eruptive seismicity and steady-state seismicity include both deep and shallow events, whereas events tend to be clustered exclusively at shallow depths immediately before eruptions (*11*–*15*). Other studies focusing on the waveform similarity of earthquakes in volcanic settings find that noneruptive events tend to exhibit more similarity and temporal regularity than events before and during eruptions, which may be interpreted as eruptions presenting a substantial change in geologic and stress conditions (*16*–*18*). The early work of Benoit and McNutt (*8*) constructed a database of volcanic earthquake swarms for the period of 1979–1989, including 191 swarms preceding eruption activity from which they determined that the duration of the swarm is correlated with the time between eruptive episodes, where longer swarm activity leads to a long interevent time of eruptions. On the other hand, noneruptive earthquake swarms found in a number of settings (*19*–*21*) have been attributed to underlying mechanisms such as arrested magma intrusions, snowmelt, and deep hydrothermal activity. In particular, the Yellowstone caldera has exhibited a number of earthquake swarms, which have been linked to fluid migration, as determined by elevated *b* values near the resurgent dome (*22*), multiplet analysis (*23*), as well as spatiotemporal evolution and focal mechanisms (*24*, *25*). The Long Valley caldera is an active volcanic system in California that has produced several notable noneruptive volcanic swarms. The most prominent of these began in 1978, where a period of relative quiescence spanning several decades was interrupted by a large earthquake swarm on the south rim, along with substantial uplift in the center of the caldera that continues episodically to the present day (*26*, *27*). The renewed seismicity and uplift have raised concerns of an impending eruption due to the inflation of the magma chamber (*26*); however, some studies have shown that the seismicity in the caldera is consistent with regional-scale tectonic processes, being on the eastern side of the Sierra Nevada mountain range (*28*, *27*). Geochemical studies suggest that no new magma is intruding into the system (*29*), while others have shown that earthquake swarms are triggered by low-viscosity, high-pressure hydrothermal fluids originating from an ancient crystallizing pluton underlying the caldera (*30*). The latter hypothesis is evidenced by a high-resolution seismicity analysis of two prominent swarms that occurred in 2014, which showed strongly fault-bounded seismicity with rapid migration rates suggesting transport of a low-viscosity fluid (*31*). Nevertheless, the question regarding the mechanism behind uplift remains open, given that fluid-driven mechanisms should exhibit episodes of subsidence as fluids escape the surface (*32*). The debate highlights the heterogeneous and enigmatic nature of volcano-tectonic systems and raises questions regarding how indirectly measured features such as seismicity may be interpreted in the context of the underlying geological processes.

To better understand the origins of seismicity in the Long Valley caldera, we reprocessed nearly 25 years of continuous seismic data, with deep-learning algorithms for earthquake monitoring (*33*–*36*). We created a high-resolution seismicity catalog and subsequently performed a cluster analysis to comprehensively quantify the spatial and temporal dynamics of seismicity within and outside of the caldera.

## RESULTS

### Overview of seismicity features

**Fig. 1:**
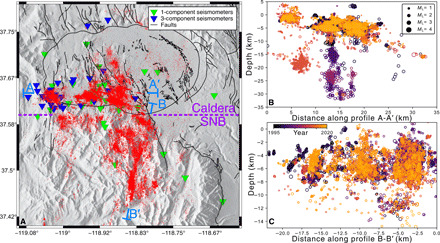
Map and cross-section views of relocated seismicity. (**A**) Map view of all relocated seismicity. Light blue bars indicate locations of depth cross sections A-A′ (5-km projection distance) and B-B′ (1-km projection distance), and purple dashed line indicates the dividing line between the caldera and the Sierra Nevada block (SNB). Black lines denote locations of known faults, including the caldera rim. One- and three-component seismometer locations are indicated in inverted triangles. (**B**) Depth cross-section A-A′ along the southern rim of the caldera. Note the base of the shallow seismogenic zone dips gently from approximately 5 km in the west to 10 km in the east. (**C**) Depth cross-section B-B′ along the seismicity in the SNB. Depths are referenced to sea level.

We present an earthquake catalog comprising 260,312 events spanning 1995–2019, including 169,638 events that were relocated with waveform cross-correlation methods (Fig. 1). The map view of seismicity demonstrates a north-south striking of the faults in the Sierra Nevada block (SNB), which transitions to east-west and northwest-southeast striking features in the caldera. The depth cross-section A-A′ indicates that the bulk of the seismicity occurs in the top 5 to 10 km, with the base of this seismogenic zone sharply delineated by a subhorizontal seismicity surface. This transition gently dips from a depth of approximately 5 km in the west to 10 km in the east, and the B-B′ depth cross section suggests that the boundary is flat lying in the north-south direction. This seismicity structure has been interpreted as being a thermally controlled brittle-ductile transition (*37*) overlying an ancient pluton consisting of crystalline mush (*30*). The A-A′ depth cross section also shows isolated clusters of earthquakes at depths greater than 15 km, which may be related to dike intrusions or fluid pressure pulses (*38*).

### Cluster dynamics

**Fig. 2:**
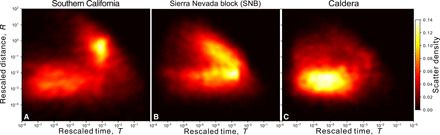
Normalized spatial and temporal distribution of NND η shown in color. Presented for (**A**) southern California (SCSN), *M*_c_ = 3, *n* = 13,044; (**B**) the SNB, *n* = 34,649; and (**C**) the caldera, *n* = 31,574.

To study the spatiotemporal behavior of seismicity over the 25-year period, we divide the earthquake catalog into clusters using a nearest-neighbor approach (*39*). This method computes a space-time distance between pairs of events and identifies the nearest neighbor (parent) for each event, resulting in a single tree structure spanning the entire catalog. The distribution of nearest-neighbor distances (NNDs) in tectonic regions for large space-time windows commonly exhibits two modes: one that occurs at short space-time distances and has been interpreted as due to clustering processes such as aftershocks or aseismic forcing, and the other that has roughly a Weibull distribution and is consistent with a Poisson process (*40*). An example is shown for the entire southern California region in Fig. 2A, where the distance is decomposed into spatial and temporal components (see Materials and Methods). For our study area, we separate events within the caldera (Fig. 2C) from those immediately outside to the south (Fig. 2B). The NND distribution for events in the latter region exhibits the two-mode pattern similar to the southern California results, corresponding to Poissonian and clustered behavior (*41*). However, the NND distribution within the caldera is markedly different and is nearly unimodal; the fraction of all earthquakes that can be viewed as triggered (not background) is an astonishing 68% compared to 34% in adjacent SNB. This indicates that the caldera earthquakes predominantly occur within sequences, in contrast to the seismicity in the SNB, which has many more background events relative to the total number that occurred. To better understand these observations, we next examine seismicity behavior of earthquake clusters.

**Fig. 3:**
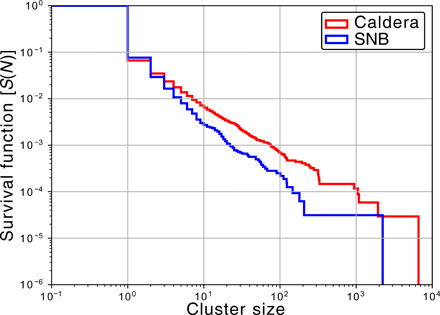
Survival function for cluster size. Presented for clusters in (red) the caldera and (blue) the SNB.

The NND diagrams provide a simple means to extract seismicity clusters by breaking the links of the spanning tree whenever the NND exceeds some threshold (see Materials and Methods). Applying this procedure results in 10,750 clusters within the caldera and 21,096 clusters in the SNB. The large number of clusters allows detailed examination of the cluster patterns between the two regions. Figure 3 shows cumulative distributions for the number of events in a cluster. The clusters in the caldera are seen to be statistically larger than those clusters immediately to the south. This observation is consistent with the general findings of Zaliapin and Ben-Zion (*42*), who noted that seismicity clusters in areas with elevated heat flow were statistically larger than those in areas with lower heat flow.

**Fig. 4:**
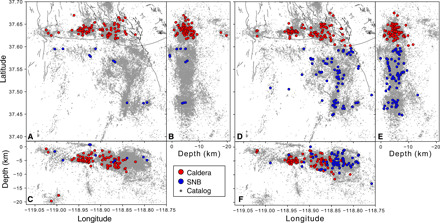
Spatial distribution of clusters with large (*d ≥* 5) and small (1 ≤ *d* < 5) mean leaf depth. (**A** to **C**) Map and north-south and east-west views of large leaf depth root event locations. (**D** to **F**) Small leaf depth root event locations.

The tree structures associated with a set of clusters have been used to study topological aspects of seismicity. Using the term leaf to describe an event with no offspring, we next focus on a quantity called the leaf depth, *d*, which is defined as the number of generations separating a given leaf and the root of the tree. The average leaf depth over the tree has been shown to be useful for distinguishing swarm-type clusters from more typical mainshock-aftershock clusters (*42*), with swarms tending to exhibit larger values, indicating that each event tends to trigger few offspring, resulting in long chain–like tree structures. Mainshock-aftershock sequences tend to have a small number of events that trigger most of the events in the sequence, resulting in trees with few generations. Figure 4A shows a map of seismicity clusters that have mean leaf depth *d* ≥ 5, while Fig. 4B separately indicates clusters with *d* < 5. More than 95% of the clusters with *d* ≥ 5 are located within the caldera directly; however, the number of clusters with *d* < 5 is similar between the caldera and the adjacent SNB. Thus, the spatial distribution of swarm-like clusters terminates abruptly at the southern rim of the caldera.

The question of whether swarms tend to migrate upward or downward is important for understanding the physical processes and conditions underlying swarm evolution. Basic considerations of the diffusion problem lead to the expectation that swarms should be upward along a hydraulic gradient [e.g., (*43*)]. The handful of well-documented swarms in the Long Valley caldera and SNB, however, have exhibited both upward (*38*, *31*) and downward (*44*) migration patterns; the limited number of examples here is too small to make any kind of general statements about swarm behavior in the caldera. Given the large number of clusters in our dataset, we can test whether there is a statistical tendency for swarms to migrate upward. We define a vertical mean migration distance $\Delta \bar{Z}=Z_{\text{root}}-\bar{Z}$ for each cluster, where *Z*_root_ is the depth of the first (root) event in the cluster, $\bar{Z}$ is the cluster mean depth, and positive $\Delta \bar{Z}$ distances indicate ascending migration. To determine whether clusters systematically exhibit ascending migration within and outside of the caldera, we conduct one-sided *t* tests with the null hypothesis that clusters in the caldera and the SNB exhibit $\Delta \bar{Z}=0$, i.e., that clusters on average do not exhibit ascending migration. The results in Table 1 show that we cannot reject the null hypothesis for clusters in the SNB. In the caldera, we can reject the null hypothesis at 10% significance in the caldera, indicating that clusters in the caldera have statistically significant tendency to migrate upward in the 25-year study period. We do note that the histogram of $\Delta \bar{Z}$ shown in fig. S6 indicates that the difference between the caldera and the SNB is somewhat subtle. The mean migration distance for the caldera is 85 m and 13 m for the SNB. The SEs are 49 m for the caldera and 115 m for the SNB. We find that of the caldera clusters, 36% exhibit ascending migration greater than 100 m, while 23% exhibit descending migration greater than 100 m. Similarly, the proportions in the SNB are 45% ascending >100 m, 33% descending >100 m. Given the location errors, it is difficult to resolve these patterns for individual clusters; however, the one-sided *t* test shows that the migrations are systematically positive in the caldera.

**Table 1:** One-sided *t* test for the expected value of migration distance $\Delta \bar{Z}$ in the caldera and SNB.

**Null hypothesis *H*_0_**	**Sample size *N***	***t* statistic**	***P* value**	**Decision**
$\Delta {\bar{Z}}_{\text{Caldera}}=0$	159	1.97	0.0255	Reject at 10% significance
$\Delta {\bar{Z}}_{\text{SNB}}=0$	78	0.0797	0.468	Accept

**Fig. 5:**
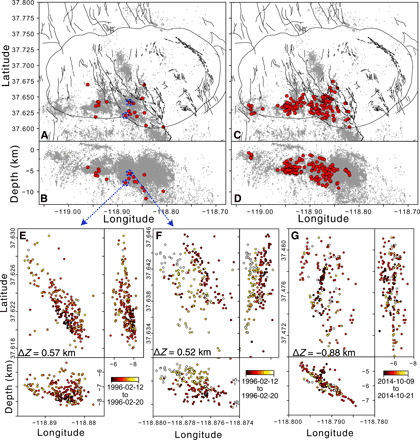
Spatial distribution of clusters with large ($\Delta \bar{Z}>0.5\;\text{km}$) and small ($\Delta \bar{Z}<0.5\;\text{km}$) mean migration distance. (**A** and **B**) Map and east-west views of large migration distance root event locations. Blue circles and arrows show nucleation points of clusters shown in (E) and (F). (**C** and **D**) Small migration distance root event locations. Note that while the nonmigrating clusters occur throughout the seismogenic zone, the upward migrating clusters occur exclusively near its base. (**E** and **F**) Examples of ascending clusters in plan and section views, where color indicates time. (**G**) Example of a descending cluster in plan and section views, where color indicates time.

Given the finding that the caldera swarms are statistically likely to migrate upward, we can examine the spatial distribution of the clusters exhibiting strong migration. Figure 5A shows the nucleation site of clusters with $\Delta \bar{Z}\geq 0.5\;m$, while Fig. 5B separately shows clusters with $\Delta \bar{Z}<0.5$. The depth cross sections (Fig. 5, B and D) suggest that the ascending clusters in the caldera nucleate near the base of the seismogenic zone, whereas non-ascending clusters are distributed across the entire seismogenic zone. This can again be systematically investigated using a one-sided *t* test, with the null hypothesis that ascending clusters ($\Delta \bar{Z}\geq 0.5\;\text{km}$) and non-ascending clusters ($\Delta \bar{Z}<0.5\;\text{km}$) have the same global mean depth (averaged over all clusters within each group). The null hypothesis is rejected with 10% significance when comparing ascending clusters in the caldera to non-ascending clusters in the caldera, as well as comparing ascending clusters in the caldera to all other clusters in our catalog (Table 2). This indicates that ascending clusters in the caldera nucleate deeper on average than the non-ascending clusters. Examples of the spatiotemporal evolution of ascending clusters are shown in Fig. 5 (E and F), and an example of a descending cluster is shown in Fig. 5G.

**Table 2:** One-sided *t* test for the mean depth of ascending ($\Delta \bar{Z}>0.5\;\text{km}$) and non-ascending ($\Delta \bar{Z}<0.5\;\text{km}$) clusters. Sample size column indicates *N* for the two populations considered. ${\bar{Z}}_{\text{remaining}}$ refers to the mean depth of the population consisting of SNB clusters and non-ascending caldera clusters.

**Null hypothesis *H*_0_**	**Sample size *N***	***t* statistic**	***P* value**	**Decision**
${\bar{Z}}_{\text{Caldera},\text{ascending}}={\bar{Z}}_{\text{Caldera},\text{non}-\text{ascending}}$	18/141	1.43	0.0778	Reject at 10% significance
${\bar{Z}}_{\text{SNB},\text{ascending}}={\bar{Z}}_{\text{SNB},\text{non}-\text{ascending}}$	16/62	0.758	0.226	Accept
${\bar{Z}}_{\text{Caldera},\text{ascending}}={\bar{Z}}_{\text{remaining}}$	18/219	1.75	0.0409	Reject at 10% significance

## DISCUSSION

**Fig. 6:**
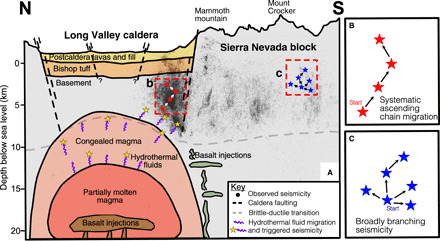
Schematic north-south depth cross section. (**A**) Earthquake locations in black circles, inferred fault lines in dashed black lines, and inferred brittle-ductile transition in dashed gray lines. Black lines denote inferred geological boundaries. (**B**) Topological representation of long chain–like earthquake clustering commonly found in the caldera. (**C**) Topological representation of tectonic-type clustering found commonly in the adjacent SNB.

Our results show that seismicity in the Long Valley caldera includes a substantial fraction of episodic swarm-type events characterized by short normalized interevent times and distances, relatively large cluster sizes, and large leaf depths denoting a long chain–like topology, where each event is linked to only a small number of subsequent events (Figs. 2C and 6B). These swarm-type clusters, which statistically exhibit a tendency for ascending migration patterns, are markedly distinct from typical tectonic clusters such as in the adjacent SNB, where individual mainshocks trigger large families of aftershocks with substantial branching (e.g., each event directly triggers many aftershocks) (Figs. 2B and 6C). In addition, the ascending swarm-type clusters occur almost exclusively within the caldera (Figs. 4A and 5A), with a sharp demarcation at the caldera rim suggesting that these swarms are specifically triggered by volcanic processes underlying the caldera (Fig. 6A). The observations further provide a line of evidence that the caldera rim is a key boundary for these volcanic processes, which may not extend beyond it.

Previous geological (*30*), geochemical (*29*), and seismological studies (*31*) provide evidence that these volcanic swarms are triggered by the transport of hydrothermal fluids originating from below the seismogenic zone, which are produced as part of the degassing of an ancient caldera-wide pluton. These hydrothermal fluids could account for the uplift seen at the resurgent dome and may be relieved by transport along steeply dipping ring faults along the south rim of the caldera. These ring faults were likely formed or activated in response to the complex tectonic stresses on the edge of the Sierra Nevada mountains (*28*, *45*), leading to elevated shear stresses that trigger tectonic-type double-couple focal mechanisms seen alongside non–double-couple mechanisms in the caldera (*46*). This elevated shear stress is also seen in our results, where the clusters in the caldera, although dominated by the short normalized interevent times and distances, nevertheless contain a number of clusters with shorter leaf depths and non-ascending behavior (Fig. 4D). The hypothesis of combined fluid-driven and tectonic stress changes may be further supported by the general lack of seismicity east of the resurgent dome (Fig. 1A), which is more distal from the stress changes induced by the Hilton Creek fault and thus less likely to form or activate faults. As a result, the resurgent dome likely consists of relatively intact material forming a central piston-type block similar to that observed at Kilauea (*47*).

We further show that swarms exhibiting large positive migration distances (ascending, $\Delta \bar{Z}>0.5\;\text{km}$) occur at greater depths than other earthquake clusters in Long Valley (Fig. 5). The root events for these ascending clusters are evenly spaced across the south rim of the caldera but are not present at Mammoth mountain on the southwest corner of the caldera, suggesting that these root event locations exclusively represent nucleation sites of hydrothermal fluid conduits originating from the ancient crystal mush. On the other hand, the seismicity under Mammoth mountain may be attributed to a different triggering mechanism, as suggested by the presence of large and small leaf depth clusters (Fig. 4) while lacking upward migrating swarms (Fig. 5A). Here, the seismicity may be more closely related to changes in an active magma chamber (*38*, *48*), which is localized to the active volcanism at Mammoth and the Mono-Inyo chain extending to the north.

Seismicity patterns globally have long been observed to exhibit extraordinary variability in their behavior, even on relatively short length scales; however, historically, it was unclear how much of this complexity was due to observational error and/or the non-uniqueness associated with geophysical inverse problems. The recent improvements to seismicity catalogs resulting from increased detection sensitivity (*49*–*51*) and picking accuracy (*52*, *53*) have repeatedly shown that such complexity appears to be genuine, especially for swarms (*3*, *43*, *54*). The highest-resolution observations of recent years have further highlighted the importance of spatially variable fault zone properties and processes in underlying such dynamic behavior. Here, we demonstrate that these enhanced catalogs, which can be readily scaled to long study periods owing to its underlying automated algorithms, can be robustly probed for long-term patterns using statistical techniques. Our results, which broadly characterize fluid-triggered and regional tectonic seismicity under complex thermal-hydro-mechanical conditions, provide a reference point for regions with sparse network coverage and/or shorter time scales. Thus, our results represent a substantial step forward in our understanding of subsurface dynamics in magmatic regions.

## MATERIALS AND METHODS

### Earthquake detection and location

Raw waveform data were downloaded from between 1995 and 2019 for stations within a box surrounding the Long Valley caldera (latitude, 37.2 °N to 38.2 °N; longitude, 119.4 °W to 118.4 °W), including the Incorporated Research Institutions for Seismology (IRIS), Northern California Earthquake Data Center (NCEDC), and Southern California Earthquake Data Center (SCEDC) data centers. The three-component waveform data were processed for P- and S-wave arrivals using the generalized phase detection (GPD) (*33*) method, which was trained using the NCEDC catalog picks. The one-component waveform data were similarly processed for phase picks using a modified GPD algorithm, which was trained with NCEDC catalog picks, where the training data consisted of 50% noise waveforms for regularization to minimize the number of false-positive phase picks.

Phase picks were then associated with the PhaseLink algorithm (*34*), where the model weights were first trained on a synthetic catalog of earthquake locations. This model was used to create an initial catalog of real earthquakes in the Long Valley region spanning 1995–2019, and then the initial catalog was used to improve the PhaseLink model weights using transfer learning. The events were initially located with the NonLinLoc package (*35*) using a three-dimensional S-wave velocity model (*55*) and *V*_P_/*V*_S_ = 1.68, and then relocated using GrowClust (*36*). The final catalog consists of 260,312 events, of which 169,638 were relocated. Magnitudes were calculated using a local magnitude scale (*56*) and corrected to a duration magnitude scale using an empirical calibration suggested by the U.S. Geologic Survey (figs. S1A and S2) (*31*). Newly detected events with *M* > 2 are not assigned a magnitude in the catalog to avoid including events with clipped waveforms. The magnitude of completeness is assessed with guidance from the maximum curvature method (*57*) using a 2000 event rolling window; we find *M*_c_ = 1.0 before 2011 and *M*_c_ = 0.5 after 2011 (fig. S3). We classify earthquakes with a latitude greater than 37.6° as originating from the caldera and latitude less than or equal to 37.6° as originating from the SNB (*28*). The regional catalog from the NCEDC covering the same period and spatial extents contains 131,912 events, of which 112,469 are also detected within our enhanced catalog totaling 255,650 events. Of the events common to the NCEDC and our catalog, 80% of epicentral locations were within 2.13 km, and 80% of depths were within 2.45 km (fig. S1B).

### Clustering

The earthquakes are divided into clusters in the space-time-energy domain using the NND formulation proposed by Zaliapin and Ben-Zion (*39*), where each event *j* is assigned a parent event *i* separated by NND η = *R_ij_T_ij_*, where normalized distance *R* is defined as *R_ij_* = (*r_ij_*)*^d_f_^*10^−(1 − *q*)*bm_i_*^, normalized time *T* is defined as *T_ij_* = *t_ij_*10^−*qbm_i_*^, *r_ij_* is the hypocentral distance in kilometers, *t_ij_* is the time between events in years, *m_i_* is the magnitude, *d_f_* = 1.6 is a fractal dimension of the earthquake hypocenter distribution, *b* = 1 is the slope of the Gutenberg-Richter frequency-magnitude distribution, and *q* = 0.5 is the relative weighting of the normalized time and distance. The method has been successfully applied to large earthquake catalogs spanning 111,981 events across southern California (*39*), as well as regional scale–induced earthquake sequences such as a catalog with around 8000 events at the Coso geothermal fields (*58*).

Within this framework, an earthquake catalog is assumed to contain two distinct populations consisting of (i) parent events demarked by large NND and (ii) child events at small NND. Given a population of η for the entire catalog, we then use a Gaussian mixture model to fit a bimodal distribution to η and determine η_c_ = − 6.2 (fig. S4) as the best-fit parameter to separate the two populations. We then formulate clusters by removing all links larger than η_c_ such that any event with η > η_c_ is considered the first, or root, event in a cluster. Any child events of the root event, and their subsequent children, are considered within this cluster. The leaf is then defined as an event without a child, with leaf depth defined as the number of links between the leaf and the root event. The branching ratio is defined as the number of children linked to a parent event.

Within each cluster, outliers are identified and removed if the event depth exceeds the inner fence and is more than 2.5 km from the median depth of the cluster. The upper and lower inner fences are defined as *Q*3 + 1.5 * (*Q*3 − *Q*1) and *Q*1 − 1.5 * (*Q*3 − *Q*1), respectively, where *Q*1 and *Q*3 are the 25th and 75th percentiles, respectively. Of the 65,656 events in the clustered catalog, 573 were determined to be outliers and removed. The survival function is calculated as *S*(*N*) = 1 − cdf(*N*), where cdf(*N*) is the empirical cumulative density function of the population of cluster family sizes.

### Migration distance

We define the migration statistic of each cluster as the parameter $\Delta \bar{Z}=Z_{\text{root}}-\bar{Z}$, where *Z*_root_ is the depth of the first event in a cluster, and $\bar{Z}$ is the mean depth of the cluster. Because depth is positive downward, a positive value of $\Delta \bar{Z}$ indicates upward migration of the cluster. We test whether clusters in (i) the caldera and (ii) the SNB exhibit upward migration using a one-sided *t* test, with the null hypothesis that the mean migration is 0.

We further define clusters with $\Delta \bar{Z}>0.5\;\text{km}$ as ascending clusters and additionally test the hypothesis that the ascending clusters systematically nucleate deeper than non-ascending clusters, again using a one-sided *t* test with the null hypothesis that the mean depths of ascending clusters are statistically indistinguishable from the mean depths of non-ascending clusters. This test is used to compare (i) ascending with non-ascending clusters in the caldera, (ii) ascending with non-ascending clusters in the SNB, and (iii) ascending clusters in the caldera with all other clusters.

## SUPPLEMENTARY MATERIALS

Supplementary material for this article is available at http://advances.sciencemag.org/cgi/content/full/7/35/eabi8368/DC1
